# TogoDoc Server/Client System: Smart Recommendation and Efficient Management of Life Science Literature

**DOI:** 10.1371/journal.pone.0015305

**Published:** 2010-12-13

**Authors:** Wataru Iwasaki, Yasunori Yamamoto, Toshihisa Takagi

**Affiliations:** 1 Department of Computational Biology, University of Tokyo, Kashiwa, Japan; 2 Database Center for Life Science, Tokyo, Japan; 3 Center for Information Biology, National Institute of Genetics, Mishima, Japan; Kyushu Institute of Technology, Japan

## Abstract

In this paper, we describe a server/client literature management system specialized for the life science domain, the TogoDoc system (Togo, pronounced Toe-Go, is a romanization of a Japanese word for integration). The server and the client program cooperate closely over the Internet to provide life scientists with an effective literature recommendation service and efficient literature management. The content-based and personalized literature recommendation helps researchers to isolate interesting papers from the “tsunami” of literature, in which, on average, more than one biomedical paper is added to MEDLINE every minute. Because researchers these days need to cover updates of much wider topics to generate hypotheses using massive datasets obtained from public databases or omics experiments, the importance of having an effective literature recommendation service is rising. The automatic recommendation is based on the content of personal literature libraries of electronic PDF papers. The client program automatically analyzes these files, which are sometimes deeply buried in storage disks of researchers' personal computers. Just saving PDF papers to the designated folders makes the client program automatically analyze and retrieve metadata, rename file names, synchronize the data to the server, and receive the recommendation lists of newly published papers, thus accomplishing effortless literature management. In addition, the tag suggestion and associative search functions are provided for easy classification of and access to past papers (researchers who read many papers sometimes only vaguely remember or completely forget what they read in the past). The TogoDoc system is available for both Windows and Mac OS X and is free. The TogoDoc Client software is available at http://tdc.cb.k.u-tokyo.ac.jp/, and the TogoDoc server is available at https://docman.dbcls.jp/pubmed_recom.

## Introduction

Recent technological advances have enabled life scientists to conduct massively parallel experiments and access an abundance of data sets publicly available on the Internet [1]. Consequently, biologists today are engaged in more research fields than ever. To generate hypotheses and interpret experimental results within this context, researchers need to (1) keep up with advancements in many fields and (2) organize and codify knowledge in distant fields. Despite recent efforts devoted to knowledge engineering technologies, such as the Semantic Web and ontologies [2], the most popular medium of such knowledge is still literature written in natural languages, much of which is available electronically today [3]. Therefore, it has become increasingly important, particularly in the life science domain, to retrieve useful knowledge from public literature databases and manage personal electronic literature libraries effectively [4].

MEDLINE, the most representative literature database in biology and medicine, continues to grow at an extremely fast pace. Over the past five years, MEDLINE's entries have increased by about 650,000 per year on average [5]; it may be worth recalling that one year only contains about 525,000 minutes. Modern researchers engaged in numerous fields must check not only their accustomed journals but also this entire “tsunami” of literature. In addition, to accomplish the goal of interdisciplinary research, it is necessary to effectively connect the knowledge retrieved from diverse literature. Papers already read should be easily accessible, even without clear intention. Researchers reading numerous papers sometimes only vaguely remember or completely forget what they read in the past. As a result, it is common that huge amounts of valuable literature are buried in personal libraries, typically as electronic PDFs on storage disks. Today's researchers are becoming increasingly busy [6], and highly efficient and time-saving literature management is in great demand. For example, one of the most cumbersome tasks in literature management is classification. Although it is common to classify papers by placing them in separate folders or by giving them “tags”, it is often difficult to create and manage good classification schemes, especially if the number of managed papers increase.

Several tools have been developed for the dual objectives of finding literature on topics of researchers' interest and managing personal libraries. For the former, a classic and still popular solution is to use the PubMed search system periodically to check for updates, a process that can be automated using the MyNCBI service [7]. CiteULike [8] adopts so-called collaborative filtering engines, whose strengths have been proved by popular applications such as Amazon.com [9]. PURE [10] is a pioneering system that adopts content-based filtering, exclusively specialized for literature recommendation and equipped with a rather simple web interface. Zotero [11], Mendeley [12], and CiteULike allow users to select favorite colleagues on the Internet to check updates in their libraries. For the latter objective, the most popular software programs, including Zotero, Mendeley, iPapers [13], and Mekentosj Papers [14], provide literature management functions. Whereas iPapers and Mekentosj Papers are MacOS X applications, Zotero (a Firefox plug-in) and Mendeley are multi-platform applications. Except for Mekentosj Papers, these tools are available free of charge.

In the present work, we first conducted a requirement analysis for both objectives. Then, following the analysis, we developed the TogoDoc server and the TogoDoc Client software (*Togo*, pronounced *Toe-Go*, is a romanization of a Japanese word for integration). These two components work cooperatively to provide life scientists with a finely tunable personalized literature recommendation service and highly efficient library management. Recent advanced computing and network technologies have proven their applicability to so-called cloud computing, by which users can easily benefit from a huge amount of computing resources that they could otherwise not use on their personal computers. The TogoDoc system takes this paradigm and provides services that need large scale computing resources, such as literature recommendation, on the server, while users need to learn little about how to access the services. At the same time, relatively high performance personal computers equipped with gigabyte memories and multi-core processors have become widely distributed. To offer the best user experience, it is a promising tactic to adopt special client programs that effectively use those resources while communicating with the server. Thus, we developed a client program that offers much better interfaces and operability than general web browsers do. The TogoDoc system is available for both Windows and MacOS X, and it is free.

## Results and Discussion

### Requirement Analysis–Recommendation

First, we analyzed the requirements for taking full advantage of the knowledge described in the literature in this era of omics sciences and the information explosion. The investigation was carried out in collaboration with both experimental and computational biologists. Approximately 30 researchers answered open-ended (free) questions either in face-to-face interviews or through emails. Some of these biologists work in small-scale molecular/cellular biology and others are in large-scale omics analysis. It was revealed that even biologists doing small-scale experiments access a number of public databases on the Internet on a daily basis, engaging information in fields distant from theirs.

The existing popular tools for finding literature of potential interest are generally based on either the Boolean search or collaborative filter techniques. The Boolean search engine has been adopted by PubMed and MyNCBI, which are services of the National Center for Biotechnology Information (NCBI) [7]. These tools require users to create queries composed of keywords and the Boolean operators AND, OR, and NOT. Although this model is very popular and has been adopted by virtually all information retrieval engines, such as Google, its shortfalls are widely known [15]. First, it is not easy to cover requisite keywords. This difficulty arises partly because a specific concept is often referred to by a number of different words and expressions (i.e., synonyms). Second, it is difficult to translate the sets of keywords into Boolean expressions. Simply concatenating them by the OR operators often results in too many hits or false positives; to avoid this problem, complex and long Boolean expressions often need to be carefully constructed. Third, because researchers' interests frequently change according to their research progress and trends in research communities, the difficult task of creating appropriate Boolean expressions needs to be continuously repeated. Finally, the Boolean model cannot rank or filter retrieved papers according to different intensities of the researcher's interest in different keywords, although appropriately sorted or filtered lists can be of great help to effectively find papers of potential interest.

The collaborative filtering engine in the academic literature domain is provided by the web-based service CiteULike [8], which works as follows. First, researchers register lists of papers to the service. Second, for each of the researchers, the service finds other researchers who register similar lists. Then, the service recommends papers that frequently appear in those lists, but not in the list of the researcher in question. This collaborative filtering enables researchers to skip the cumbersome process of creating complex Boolean expressions or finding people to check their updates. However, this technique has also some drawbacks. First, the collaborative filtering tends to select popular papers appearing in famous journals and miss ones that are in minor journals but that are in the right niches for each researcher's interest. Second, the technique uses only the presence/absence of papers in its recommendation and ignores the aspects in which researchers are interested. If a user is interested in a particular part of a paper (e.g., the methods section) and most of the other users are interested in another part (e.g., the results section), papers related not by the methodology but by the findings are likely to be recommended. Finally, and above all, the recommendation of papers already focused on by many people is in opposition to the notion that researchers are striving to be unique.

In summary, the requirements for literature recommendation include (1) high efficiency, (2) recommendation scores or rankings that reflect user aspects, and (3) consideration of literature content.

### Requirement Analysis–Literature Management

For the management of personal electronic PDF literature libraries, the following requirements were identified. First, management should be conducted as efficiently as possible. PDF files should be analyzed and organized automatically, and cumbersome tasks such as classifying documents, placing the appropriate tags, and renaming file names should be kept to a minimum. Moreover, because many researchers have already collected abundant PDF papers in their storage disks, such a collection of documents, in addition to those gradually added after the system installation, should be easily processed. Second, the libraries should be synchronizable among different computers. Researchers often use several computers in parallel, so their libraries, including downloaded PDF files, should be accessible from several machines. Third, the system should provide various ways to access the “buried” literature. These would include methods that enable researchers to access papers that they do not explicitly search for, in addition to the common methods such as the full-text search, metadata search, and tag search. A promising technique is the associative search, which has been successfully adopted by the PubMed *related citations* service to complement its Boolean search function [16]. Fourth, the system should run on different operating systems, typically both on Windows and on MacOS X. Finally, it would be better if it were provided free of charge.

### Client Installation

**Figure 1:**
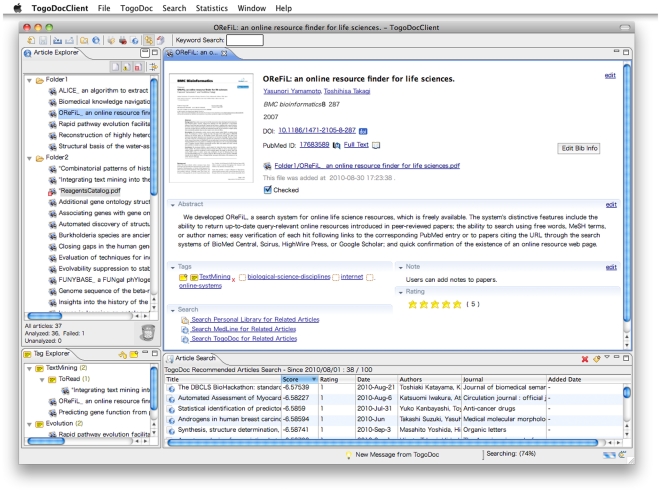
Screenshot of TogoDoc Client. The four panes are, clockwise from the upper left pane, Article Explorer, Literature Tabs, Search Pane, and Tag Explorer. Article Explorer is a file manager for all PDF-storing folders specified by users, which are *Folder 1* and *Folder 2* in this example. The filenames of PDF papers are listed, and many functions are available via right-click context menus. The icon to the left of *ReagentsCatalog.pdf* indicates that this file could not be analyzed by the Client, and the asterisks before the filenames indicate that these files have not yet been checked by the user; they are preferentially displayed at the top of the file lists. Literature Tab presents detailed information about papers selected in Article Explorer or Search Pane, and it also lets users edit their bibliographic information and invoke search functions. Users can open multiple tabs to check several papers simultaneously if necessary. Search Pane displays the results of the various search functions and literature recommendations. In this view, the Client is downloading a recommended paper list from the server. Tag Explorer manages tags created by users. All papers having tags are displayed in this pane as leaves of tag hierarchies. Users can create new tags in this pane in addition to Literature Tabs and can tag papers by dragging and dropping them from the Article Explorer described above. In addition, the various buttons on the top of the window and the menu bar are provided for the important and full functions of the Client, respectively. This screenshot was taken using MacOS X, and the interface is almost identical in Windows, except that the menu bar is included in the window. See main text for details of each pane.

To set up the system, only three steps are required: (1) install the TogoDoc Client on personal computers; (2) tell it which folders users normally use to save PDF papers; and (3) register an OpenID account to the Client. Then, the Client automatically begins to analyze all PDF papers within the specified folders and synchronize the data to the TogoDoc server using the OpenID account as an identity. A screenshot of the Client program is shown in Figure 1.

The Client program is distributed as zipped packages. When unpacked, a folder containing an executable file appears (an .exe or .app file in the Windows or MacOS X versions, respectively). Simply double-clicking this executable file launches the Client. Then, clicking the “Select New PDF Folder” button opens a folder selector dialog, and users can tell the Client where they usually store their PDF papers. An OpenID can also be easily registered by clicking a shortcut button and filling in boxes. OpenIDs issued by several major providers can be adopted, but the Client can automatically login to the server if the ID is a DBCLS OpenID (DBCLS stands for DataBase Center for Life Science). Otherwise, a browser window of the OpenID provider website opens for a user to manually certify the ID. DBCLS OpenIDs can be obtained at http://openid.dbcls.jp/ (also accessible via the TogoDoc menus of Client). Since all program and data files are saved under the unpacked folder, users can uninstall the Client just by removing that folder from their computers.

### Automatic Analysis of PDF Documents

The Client automatically analyzes all PDF files stored in the specified folders and their subfolders. First, for each PDF file, the Client extracts all text contained within the file and creates a thumbnail image of the first page using the JPedal library [17]. Then, the Client attempts to retrieve the paper's metadata (i.e., titles, authors, journal names, volumes, issues, page numbers, abstracts, PubMed IDs, and full-text links to the publishers' websites). If the Client finds Digital Object Identifiers (DOIs) [18] in the extracted texts, it sends them to the TogoDoc server. The server retrieves the metadata using the MEDLINE database, which contains the DOI information, and sends them to the Client. DOIs are character strings used to uniquely identify electronic documents (e.g., “doi:10.1371/journal.pone.0000000”) maintained by the International DOI Foundation. DOIs are given to most recently published academic papers and are printed on PDF papers. However, it can also be the case that the library contains old papers without DOIs or that the Client fails to find DOI strings in the extracted texts. In this case, Client sends the PDF files to the server to obtain their metadata. Then, the server analyzes their content to obtain the bibliographic information by taking the following steps. First, the server adopts an internally developed search system that searches a given text for paper titles stored in MEDLINE at high speed. If it fails, the next step is to use BibGlimpse [19], which finds bibliographic information such as titles and author names in the extracted text and looks up bibliographic entries in PubMed using them as queries.

In addition to metadata retrieval, the extracted text is used for the full-text and associative search functions of the personal libraries (see Associative Search for details). The thumbnail images are used for intuitive literature management via the graphical interface of the Client (Figure 1). The visual appearance or layout of the first pages often serves as an effective reminder of paper content. A related approach is adopted by the file managers of recent operating systems, which show small images of file content instead of classic icons whose appearances are determined by filename extensions.

### Client Interface

The Client interface contains multiple panes (Figure 1). Clockwise from the upper left pane, the panes are Article Explorer, Literature Tabs, Search Pane, and Tag Explorer. Users can change the positions of the panes freely according to their preferences by dragging and dropping the tabs. In addition, various buttons and the menu bar are provided for the important and full functions of the Client, respectively.

Article Explorer works as a file manager for all PDF-storing folders specified by users (*Folder 1* and *Folder 2* in Figure 1). Even if these folders exist at different paths in the file systems, they can be managed together in this pane. Users can open/delete/move/rename PDF files, launch web browsers to visit the PubMed-entry pages or the publisher websites, upload PDF files to the TogoDoc server, and invoke the associative search functions against the personal library and PubMed/MEDLINE. Some of these functions can be carried out by selecting folders or multiple papers, e.g., to find papers related to a collection of multiple papers or to upload many files at once. The small icons next to the PDF papers indicate their analytical status. For example, the automatic analysis fails for PDF files that are not biomedical papers but are contained in the specified folders. In this case, the files are given the “analysis failure” status (see *ReagentsCatalog.pdf* in Figure 1). Users can make such files hidden in the interface using the filtering buttons at the top of Article Explorer. Users can also filter any set of files by specifying file name patterns. Asterisks before the file names indicate that they have not yet been checked by the users, and they are preferentially displayed at the top of the file lists.

Literature Tab is the largest pane, and it presents detailed information about papers selected in Article Explorer or Search Pane. The thumbnail image, title, authors, other bibliographic information, PDF file link, abstract, tags, note, rating, and search menus are displayed here. Users can edit the metadata manually or semi-automatically by specifying a DOI or PubMed ID, and they can add/edit tags, notes, and ratings. Clicking the PubMed ID or the full-text link launches web browsers to PubMed or the publisher website, respectively. Clicking the PDF file link invokes a PDF viewer to read the paper's full text. When the tags are clicked, papers having the same tag appear in Search Pane (tag-search function). The associative search functions can also be invoked from this pane. Users can open multiple tabs to check several papers simultaneously if necessary.

Search Pane displays the results of the various search functions. These include the full-text and metadata searches for personal libraries, the PubMed search, the tag search, the associative search against personal libraries, the associative search against PubMed/MEDLINE, and, as shown in Figure 1, literature recommendation. The results are presented as tables, and the users can sort them by scores, titles, publication dates, and other criteria. When the search is conducted against PubMed or MEDLINE, the results usually include entries that are not in the personal library. In this case, these entries are indicated by different icons. If these entries are selected, only their metadata are displayed, and no thumbnail images, notes, or tags appear in Literature Tab.

Tag Explorer manages tags created by users. All papers having tags are displayed in this pane as leaves of tag hierarchies (each tag can have papers and tags as its children, as in Figure 1). The same papers can appear several times in the hierarchy if they have multiple tags, as in the so-called smart folders adopted by several applications (e.g., Apple's iTunes). Users can create new tags in this pane in addition to Literature Tabs, and they tag papers by dragging and dropping them from Article Explorer. The associative search functions against personal libraries and PubMed/MEDLINE can also be invoked from here. Thus, users can select papers having the same tag in a bunch and search for papers related to the union of that paper set. This function is also useful for finding papers that should have the same tag, but do not.

### Associative Search

The associative search function searches for papers sharing words with selected papers, and it sorts them in order of relevance. When the search is conducted against the personal library, the similarities are calculated as cosine values between the stemmed term vectors of full text weighted by Inverse Document Frequency (IDF) scores [15]. The IDF scores, which put more weight on rare terms than popular terms, are calculated using all of the MEDLINE abstracts. By virtue of this function, users can easily find papers on topics that are similar to those of the selected papers, even if they forget their existence.

When the associative search is conducted against PubMed/MEDLINE, the server returns the results using the same function as that of the literature recommendation system, which retrieves a subset from a set of papers (target set) where papers in the subset bear relevance to another set of papers (seed set). The target set comprises all the entries in PubMed/MEDLINE or their subsets that were published after a given publication date, where this date can be designated by the user. For recommendation, the default seed set is the entire collection of papers registered by users, and we assume that it reflects users' research interests well. However, any set of papers can be made a seed set for the associative search.

In general, a word set contained in a specific paper cannot sufficiently reflect a researcher's interest. Because of this limitation, using a single entry for the associative search, as the *related citations* function of PubMed [16] does, would tend to result in lower accuracy in collecting papers that the user would be interested in. Instead, the TogoDoc system accepts multiple papers to invoke the associative search and recommendations, as the effectiveness of this approach was proved [10]. For the associative search, the selection can be made by clicking one-by-one or collectively using folders or tags. Thus, for example, users can give tags to a set of papers of particular interest and use the tag-based associative search function to effectively collect papers of potential interest.

### Literature Recommendation

Literature recommendation is automatically conducted when the Client is launched and connected to the Internet. This function can also be manually initiated from a button or the menu bar of the Client by specifying the publication dates of papers to be recommended. Then, the Client requests the server to send a recommendation list of recently published papers and displays it in the Search Pane (Figure 1). Since we assume that there are mainly two types of usage, the recommendation engine consists of two content filtering components (the “Recent” and “Whole” engines). The Recent engine obtains recommendations from papers published and registered in PubMed in up to the three most recent months, and it updates the index every day to recommend the latest prospective publications of interest. The Whole engine is for older papers or the whole MEDLINE database, and it is updated every week. The Recent engine makes recommendations by calculating how papers share their “key terms”, which are automatically extracted from titles and abstracts along with author names and research topics that are assigned to each journal. The Whole engine uses MeSH (Medical Subject Headings) terms [7], [20] in addition to those key terms. MeSH is the National Library of Medicine's (NLM) controlled vocabulary used for indexing articles for PubMed/MEDLINE, and it provides a consistent way of retrieving information that may use different terminology for the same concepts.

There are two reasons we developed these two systems separately. First, there could be two representative situations in which a user wants to invoke the recommendation function, i.e., to check recently published papers in the user's research area and to survey, for example, literature related to a set of papers that the user recently read after being involved in a new field. We needed to make the system suitable to both situations. Second, it could take from a few weeks to a couple of months before MeSH terms are added to PubMed/MEDLINE entries (90 days on average) [21] because their annotation is done manually. The use of MeSH terms for recommendations has the advantage of utilizing qualified terms annotated by experts in biomedicine. However, many researchers want to be informed as soon as possible if a paper of possible interest is published. The Recent engine uses titles, abstracts, author names, and journal type data but not MeSH; therefore, it can handle recently published papers by following the daily updating policy. The Whole engine, on the other hand, fully utilizes MeSH terms, and it provides more effective recommendations for the whole MEDLINE data set.

### TogoDoc Server

**Figure 2:**
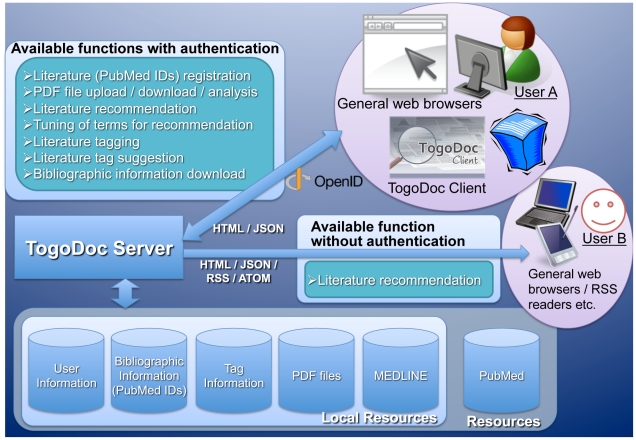
Overview of TogoDoc Server Functions. Functions available to users with or without signing in are enumerated. There are two users, A and B, that each represents a typical use of the TogoDoc system. User A uses a TogoDoc Client or a TogoDoc server web page where the provided functions can be fully utilized after signing in using an OpenID. User B uses a general web browser or an RSS reader where literature recommendations can be obtained without signing in. The ways of accessing data with their formats and resources used in the TogoDoc server are also illustrated.

**Figure 3:**
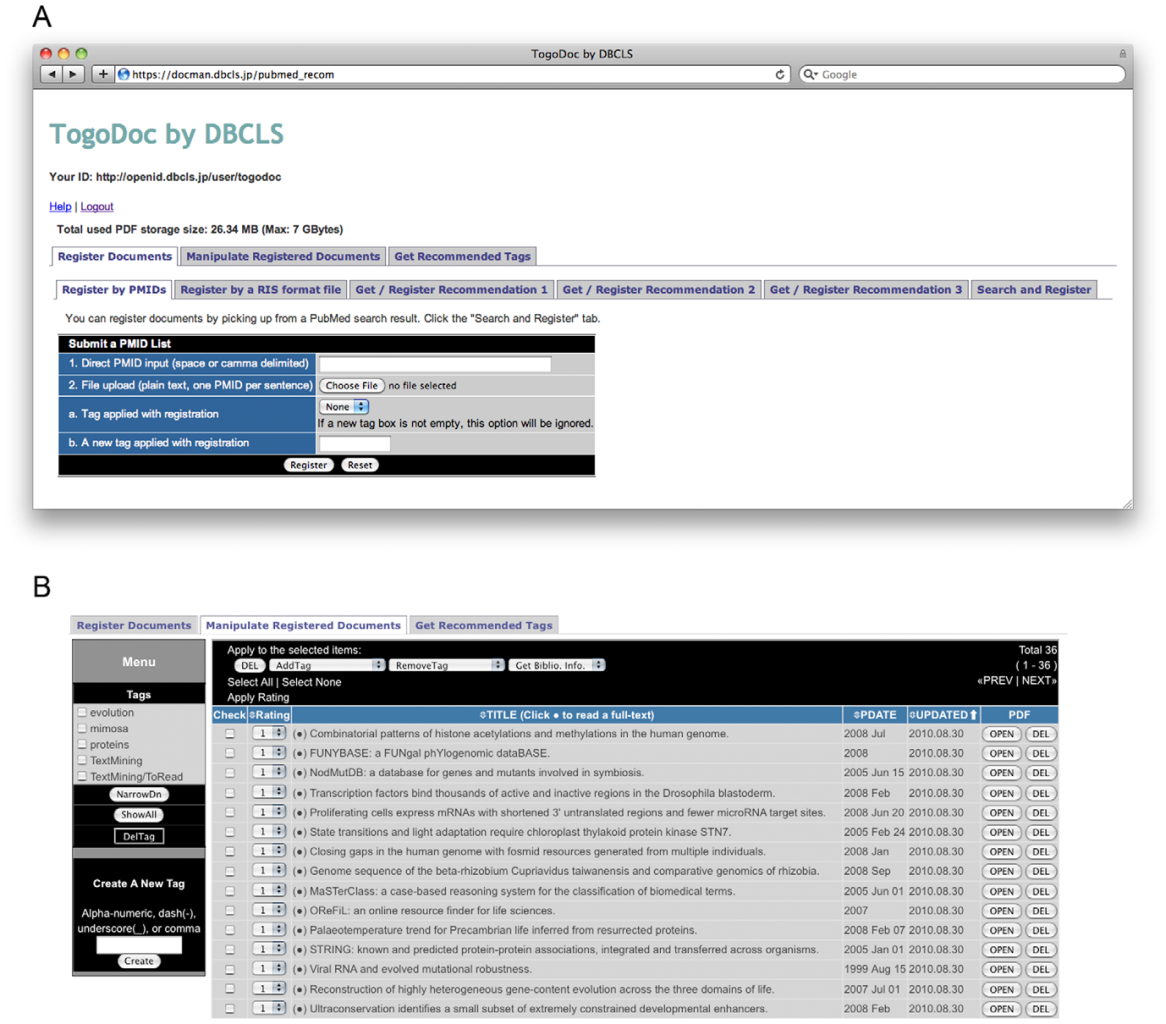
Screenshots of the TogoDoc Server Website. (A) The main page of the TogoDoc server website. A hierarchical tab-based layout is adopted where each tab corresponds to a specific function. (B) Personal library on the server website. The literature data, including the PDF files, can be registered via the website or uploaded by the Client. Users can download PDF papers by clicking the “OPEN” buttons on the rightmost column. Bibliographic information in various formats can be downloaded via the "Get. Biblio. Info." combo box just below the tabs.

The TogoDoc server provides several literature management functions, including storing PubMed-indexed literature entry lists, storing PDF files, recommending papers, and adding tags to entries. Figure 2 illustrates an overview of the TogoDoc server functions. In addition to the TogoDoc Application Program Interface (API), which is accessed by TogoDoc Client, these functions are available via a web-based interface. Through this web-based interface, users can use extra functions that are not available via Client and the API. These include tuning key terms used for recommendation and downloading bibliographic information in several formats. After users sign in to the server website, a main page shows up in the browser (Figure 3A). A hierarchical tab-based layout is adopted where each tab corresponds to a specific function. In the following, we explain these functions and interfaces.

#### 1. Literature Registration/PDF Upload

Users can register the PubMed ID lists of their library via the website in multiple ways: by inputting lists of PubMed IDs in the box, by uploading files that contain PubMed ID lists, by uploading RIS-formatted files, and by using PubMed search and recommendation results. Paper lists registered via either the website or the Client appear in a table, allowing researchers to access their personal library from any computer connected to the Internet (Figure 3B). Users can upload and download a PDF file for each bibliographic entry, a function that is seamlessly integrated with the Client: PDF files uploaded from the Client can be downloaded from the server's website and vice versa (the “PDF” column in Figure 3B). The server can analyze PDF files to extract bibliographic information and associate it with its corresponding entry, although for now, this function is only available via the API.

#### 2. Recommendation

**Figure 4:**
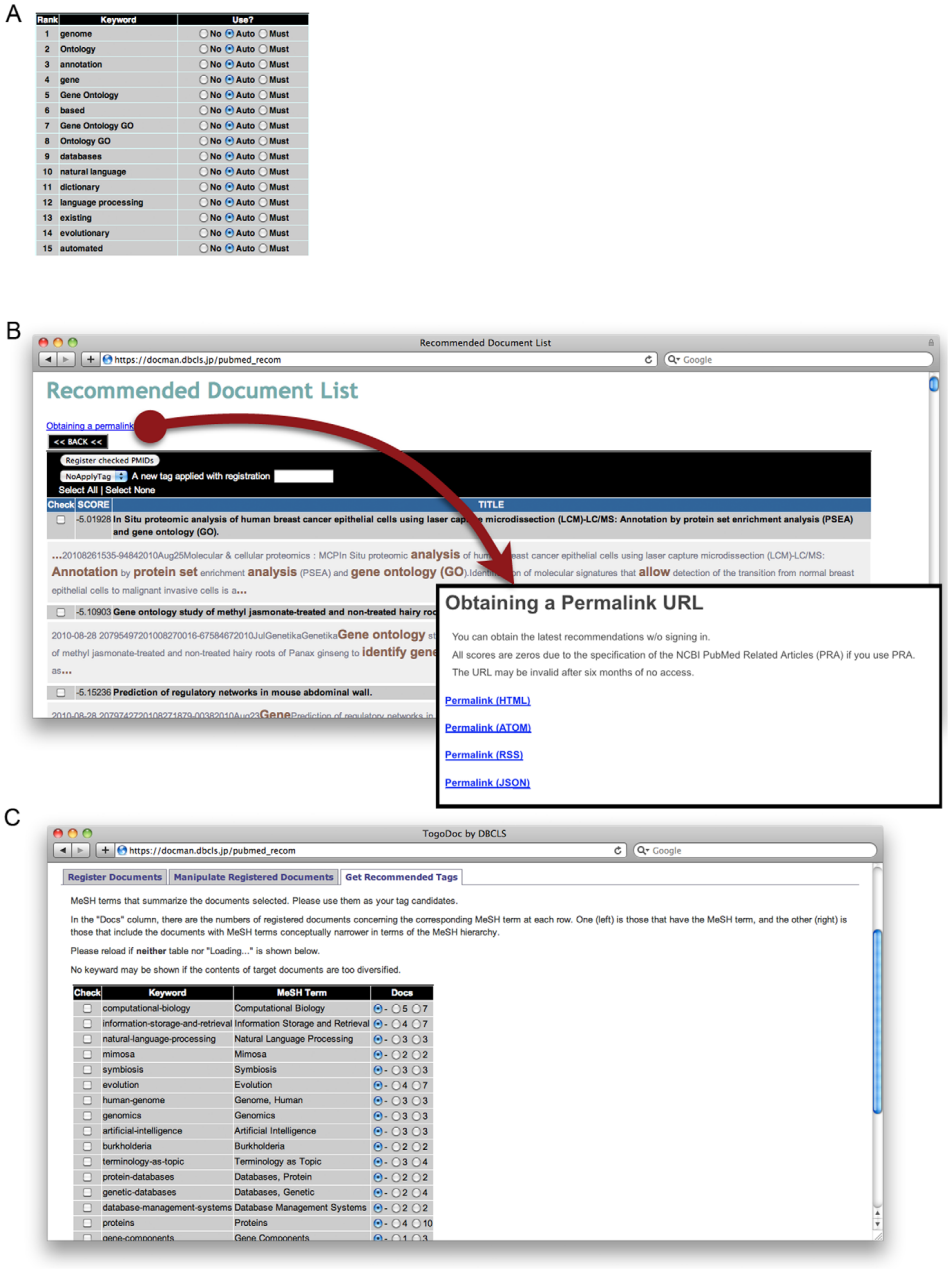
Tunable Recommendation and Tag Suggestion Functions at Server Website. (A) Tunable recommendation function on the server. Users can set some terms as stop-words and must-words that are ignored or required in recommendation, respectively. (B) A recommendation result on the server. Users can see key terms emphasized in each recommended entry (gray and brown words). A permalink for getting recommendation lists on the same condition without signing in is provided at the top of this page. (C) The tag suggestion function on the server. Suggested tag names are presented with MeSH terms on which they are based. Just a few mouse clicks are required to put these tags on papers.

The recommendation system is also available on the website, and users can optimize it by creating optional seed sets according to user-specified tags to reflect their specific interests. In addition, while the recommendation system automatically extracts key terms from the titles and abstracts, users can “tune” these terms by, for example, setting certain terms as stop-words that are ignored in the recommendation (Figure 4A). By virtue of this function, users can make the recommendation engine reflect their intensity of interest based on different key terms and the aspects how they are interested in those papers. When users invoke the recommendation function on the website, they can see each recommended entry with its key terms that are used in the recommendation (Figure 4B, gray and brown words). This function is important for improving the reliability of recommendation systems [22]; users can easily understand why the documents are recommended and can tune the key terms accordingly.

For convenience, the server provides a permalink for obtaining recommendation lists without signing in (box at the lower right of Figure 4B). This link has an encrypted number that keeps a third party from knowing about the user and the seed sets. Recommendation lists can be obtained in HTML, RSS, ATOM, or JSON formats at any time. The server processes requests on the fly, always making recommendation lists based on the latest data sets.

#### 3. Document Tagging/Tag Suggestion

Researchers often have multiple research interests, and they want to classify papers into multiple categories according to certain aspects. The TogoDoc server provides a way for users to add tags to a bibliographic entry to make literature management more efficient. While users can make any tag and add it to any set of entries, the server has a function for automatically grouping papers and suggesting tag candidates (Figure 4C). This function eases the tasks of grouping papers and considering appropriate tag names to represent the group from scratch (the tag suggestion can also be made for any arbitrarily grouped papers). Candidate tag names are based on the MeSH terms added to the papers (see Personal Library Management for details).

#### 4. Bibliographic Information Download

It is sometimes important for users to obtain bibliographic data in various formats, especially when they wish to create citation lists for their manuscripts or export them to be used by other software. Users can obtain bibliographic data in major formats, such as RIS, EndNote, Word2007, or BibTex, via the website (Figure 3B, “Get Biblio. Info.” combo box).

### Personal Library Management

The Client offers convenient management of personal literature libraries. What users basically need to do is save PDF papers to the folders that are recognized by the Client. PDF files added to the specified folders are automatically detected, analyzed, and synchronized to the TogoDoc server. Client can also be used off-line, in which case the synchronization and analysis are conducted when it is connected to the TogoDoc server. By virtue of the server API for synchronization, the Client synchronizes the data, including PDF files, to the server, and users can use multiple Clients from different computers in an integrated manner without manually copying the data.

According to the requirement analysis, one of the most cumbersome tasks in library management is the appropriate classification of papers. This includes creating appropriate categories, giving them unambiguous and intuitive names, selecting an appropriate set of tags to apply to the papers, and managing and updating the tag scheme continuously. To ease these obstacles, the TogoDoc system automatically suggests tags for its users. Tag candidates are generated based on MeSH terms assigned to papers. For each MeSH term, we can obtain a hypergeometric distribution for the entire MEDLINE data set. If a MeSH term appears frequently in a given set of papers according to that distribution, a suggested tag name is made from the MeSH term by changing the word order and making all the letters lowercase to improve its legibility as a tag. The suggested tags appear in the Client with open marks in Literature Tabs, and users can adopt these tags just by clicking them (Figure 1, Literature Tab). The associative search function against personal libraries also eases the labor of library management. Even if users add no tags to the papers, they can easily find papers on topics similar to the one of focus by using this function.

**Figure 5:**
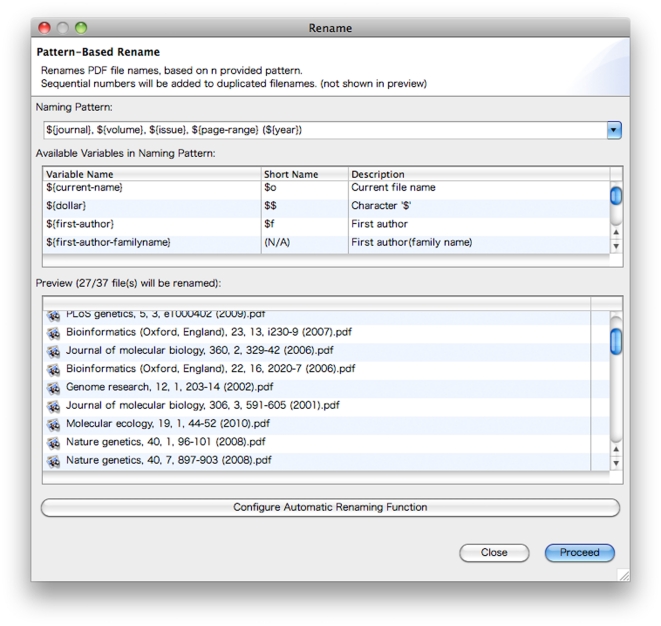
Automatic Renaming of PDF Papers by the Client. The Client can rename PDF papers that were originally saved in diverse naming styles in a batch. Uses can specify any naming pattern by using the wild cards starting with “$”. In the preview window, users can guess how the filenames of the PDF papers in their libraries will change according to the designated pattern. Clicking the *Configure Automatic Renaming Function* button at the bottom makes the Client automatically apply this filename style to newly added PDF papers to the folders managed by the Client.

The Client is equipped with further functions for convenient library management. One of these functions is automatic file renaming. When downloaded from publisher sites, the names of PDF papers are usually in highly diverse formatting styles (e.g., “1234.pdf”, “fulltext.pdf”, and “ABCD-11-1-1234.pdf”). Client can automatically rename these files into unified formats like “LiteratureTitle.pdf”, “Year-JournalName.pdf”, and “AuthorName-Year.pdf”. Users can adopt any format that can be specified as patterns according to their preferences (Figure 5). The statistics function indicates which authors and terms significantly appear in the selected papers. Using this function, users can find potential reviewers and emerging topics in particular research areas efficiently.

### Limitations

Despite the functions described above, the TogoDoc system has several limitations. First, it does not adopt literature databases other than PubMed/MEDLINE. Papers not stored in PubMed/MEDLINE are neither given metadata automatically nor synchronized to the TogoDoc server, although the full-text and thumbnails are actually extracted by the Client for some management functions, such as the associative search against personal libraries, the full-text search, and the graphical interface. This restriction exists mainly because the system aims to help researchers in the life science, although other reasons include the system taking advantage of the rich MeSH annotations. Second, although PDF papers without DOIs can also be identified by the system, those that are protected or scanned cannot. The former problem cannot be solved by computer programs in principle, but the latter may be solved by adopting optical character recognition techniques. Third, the Client does not currently accompany Microsoft Word plug-ins for insertion of citations directly into manuscripts. This is primarily because we focused on the problem of literature recommendation and management; nonetheless, bibliographic data can be obtained in several major data formats as already described.

## Materials and Methods

### TogoDoc Server

The TogoDoc server was deployed using the so-called LAMP (Linux, Apache, MySQL, and Perl) open-source software. The server runs on a Linux operating system, and the Apache HTTP Server [23] provides the web services. A MySQL database system [24] stores various data such as papers or tag data needed to realize the TogoDoc services. Perl [25] processes all of these data and the requests from users via Client or web browsers. All of these resources have been extensively used and are well supported. In addition to these, we use a database manager Tokyo Cabinet [26] to store PDF files. To provide PDF analyses, bibliographic data processing, or literature recommendation, we used several existing programs.

In the following, we explain technical issues related to server implementation.

#### 1. OpenID

The server provides personalized services, and it requires its users to obtain an OpenID. OpenID is a new authentication protocol that allows a single account to be used by multiple services and requires authentication irrespective of the service providers [27]. This protocol is beneficial for both users and service providers like us. For users, once the OpenID accounts have been obtained, they do not need to obtain others to use other services if they allow OpenID authentication. For providers, once a publicly available OpenID client module for the system has been devised, there is no need to prepare an authentication system or handle sensitive personal data such as passwords or email addresses.

#### 2. Data Exchange

To provide multiple channels for users to access their bibliographic data, the TogoDoc server adopts data transfer formats such as RSS (RSS 0.91, [28]), ATOM [29], and JavaScript Object Notation (JSON, [30]) in addition to HTML. RSS and ATOM are web feed formats that are now widely used by websites that frequently update their content, such as news sites or blogs [31]. The server uses these formats to feed the latest literature recommendations. JSON is a text format for the serialization of structured data that was derived from the ECMAScript Programming Language Standard. As its design goals include being minimal and portable, we adopted it as the data format for responses to Client requests.

#### 3. PubMed/MEDLINE

PubMed/MEDLINE data are the key contents of the TogoDoc system, especially for realizing the literature recommendation. The Recent engine needs daily updated PubMed data, which are obtained using NCBI E-utilities [32]. The data are in an XML format and are indexed by the system along with the metadata of PubMed IDs, titles, authors, index dates, and journal types (Journal Subject Terms). The Journal Subject Terms are assigned by NLM to MEDLINE journals to describe the journals' overall scope, and the Recent engine utilizes them to calculate users' research interests, giving more weight to literature within the same scope when making recommendations. MEDLINE data are used for the Whole engine, and the daily update data are obtained from NLM under the license agreement between NLM and DBCLS.

#### 4. Recommendation System

The Recent engine employs an open-source information retrieval (IR) toolkit called Lemur/Indri [33]. Recommendation is realized by issuing an automatically constructed query to the IR system, which searches for papers registered on PubMed in up to the three-month period preceding the query. The Whole engine was developed by a private company at our request, and therefore its details are not published. Here we explain how the Recent engine works.

#### 4.1. Key Term Extraction

Given a set of PubMed IDs for papers of interest to a user (an initial document set), the Recent engine extracts the key terms from their titles and abstracts, which are then used as a query to the IR system. To allow multiple words in a key term, we constructed a dictionary of N-grams from the whole MEDLINE data, where N is a number from one to five. The dictionary also contains the appearance frequency of each N-gram. We use a hypergeometric distribution to obtain the significance level of a key term; that is, for each key term appearing in the initial document set with a frequency greater than the expected frequency based on the whole MEDLINE database, the Recent engine calculates the occurrence probability of that number of times the key term appears. Then, the lowest *k* key terms are used as a query (*k* is variable and currently limited to 75).

#### 4.2. Author and Journal Data Extraction

In addition to the extracted key terms, data regarding the author and journal are also used to construct a query to the IR. As mentioned above, the Recent engine converts each journal title to a term based on its scope to effectively utilize it as the user's research interest; the author's names are simply obtained from the MEDLINE database. A researcher is assumed to regularly read a set of journals related to his or her research interest, and to submit manuscripts to journals in that set. To map each journal to its closely related research fields, we use Journal Subject Terms.

#### 4.3. Query Construction

After obtaining the key terms and the author and journal data, the Recent engine constructs a query to the IR system. The Lemur/Indri toolkit enables construction of a structured index, and the Recent engine issues a query that effectively reflects several contexts such as authors' names and journals' scope. A constructed query can be represented in natural language as follows: “Find papers that contain at least one of the given key terms and sort them in order of significance, assigning more significance to those that contain more key terms with greater weight. Also, assign greater significance to papers whose authors are in the given author list as well as those published in journals whose scope is in the given scope list.” The weight of a key term reflects the hypergeometric distribution information obtained. In addition, the key terms that appear in papers' titles are given greater weight than terms that appear in abstracts.

#### 5. API

As mentioned above, the TogoDoc server provides an API that provides recommendations to the client and synchronizes the tag data and the PDF files. All the requests from the client to the server are represented as URLs and are issued using the HTTP GET command—except for those that send a PDF file or a PubMed ID list, which are issued using the HTTP POST command. All the responses from the server, except for those that transfer a PDF file, are in the JSON format. The API's specifications are in the Supporting Information File S1.

#### 6. Other

For converting bibliographic data, we use Bibutils [34].

### TogoDoc Client

The TogoDoc Client was developed in Java as an application on the Eclipse Rich Client Platform (RCP) version 3.4.1 [35]. This is an RCP derived from the Eclipse project, which is widely known for its integrated development environment (IDE) adopted by many programmers to develop programs in various languages such as Java, C, C++, Python, Perl, and Ruby. The RCP is a compact version that serves as an application platform that simply contains toolkits useful for implementing applications, such as those for user interfaces and file managers. By adding “plug-ins” written in Java, any application can be built on the RCP. In addition to our original Java plug-ins, the JPedal library for analyzing PDF files was adopted to implement the Client [17]. The Client is updated semi-automatically by downloading additional plug-ins that are also managed by the RCP toolkits. Plug-ins developed by third parties can also be added to enhance the functions of the Client. The program can potentially run in any environment where Java version 5 or above is installed, and the current Windows and MacOS X versions whose interfaces are optimized for each operating system are distributed. TogoDoc Client is licensed under the BSD license, and its source code is available at SourceForge.JP, http://togodoc.sourceforge.jp/.

## Supporting Information

File S1 (DOC)
