# Supplementary material for: TogoDoc Server/Client System: Smart Recommendation and Efficient Management of Life Science Literature
Source: PLoS One. 2010 Dec 13;5(12):e15305. doi: 10.1371/journal.pone.0015305 (PMC3001491; doi:10.1371/journal.pone.0015305)
Supplement: File S1 — (DOC) [file pone.0015305.s001.doc]

**Supporting Information of**

**TogoDoc Server/Client System: Smart Recommendation and Efficient Management of Life Science Literature**

Wataru Iwasaki^1*†^, Yasunori Yamamoto^2*†^, and Toshihisa Takagi^1,2,3^

**1** Department of Computational Biology, University of Tokyo, Kashiwa, Chiba, Japan

**2** Database Center for Life Science, Bunkyo-ku, Tokyo, Japan

**3** Center for Information Biology, National Institute of Genetics, Mishima, Shizuoka, Japan

* E-mails: iwasaki@k.u-tokyo.ac.jp and yy@dbcls.jp

^†^ These authors equally contributed to this work.

**Supporting Text**

In this document, the API specification of the TogoDoc server is presented.

Endpoint https://docman.dbcls.jp/pubmed_recom

All the responses are in the JSON format except for obtaining a PDF file.

**1. Sign-in (Authentication)**

**iwt-openid-url=<OpenID>**

* Return

**{**

**"ErrorCode":0,**

**"Key":"<Key>",**

**"Access":"<URLforAuthentication>"**

**}**

Unless already signing-in, authentication at an OpenID provider is required, and users need to access to the URLforAuthentication.

Once authentication has successfully done, Key is used for every conversation between the server and the client.

* If a given OpenID is invalid

**{**

**"ErrorCode":200,**

**"Reason":"Invalid ID: <ErrorMessageFromOpenID_ClientModule>",**

**"Error":"<GivenOpenID>"**

**}**

**2. Sign-out (Log-out)**

**iwt-logout-key=<Key>**

* Return

**{**

**"ErrorCode":0,**

**"Accepted":"<Key>"**

**}**

* If a given key is invalid

**{**

**"ErrorCode":201,**

**"Reason":"Given key is invalid.",**

**"Error":"<Key>"**

**}**

**3. Various Commands**

**iwt-command=<CommandName>&Key=<Key>&<Options>**

**Common responses in the case of an error**

* If a problems concerning databases in the server occurs

**{**

**"ErrorCode":100,**

**"Reason":"<DBI error message or 'DB error'>",**

**"Error":"<Key>"**

**}**

* If a given Key is invalid

**{**

**"ErrorCode":201,**

**"Reason":"Given key is invalid. (It may be expired by logging in using a web browser or another PC.)",**

**"Error":"<Key>"**

**}**

* If a given command is invalid

**{**

**"ErrorCode":300,**

**"Reason":"Command Not Found",**

**"Error":"<Key>"**

**}**

* If an argument is missing or excessive

**{**

**"ErrorCode":301,**

**"Reason":"<Various messages depending on the reason>",**

**"Error":"<Key>"**

**}**

* If a given argument is invalid

**{**

**"ErrorCode":302,**

**"Reason":"<Various messages depending on the reason>",**

**"Error":"<Key>"**

**}**

* If a given file format is invalid

**{**

**"ErrorCode":303,**

**"Reason":"<Various messages depending on the reason>",**

**"Error":"<Key>"**

**}**

Below is a list of valid CommandNames.

**3.1. get-latest-server-news**

* Description: get the latest server news (this function doesn't save history).

* Option: none

* Return

**{**

**"ErrorCode":0,**

**"message":"<Message>"**

**}**

**3.2. add**

* Description: register given bibliographic data.

* Option: **pmids=<PubMed ID>,<PubMedID>,...,<PubMed ID>**

* Return

**{**

**"ErrorCode":0,**

**"Done":"<Key>"**

**}**

**3.3. delete**

* Description: delete given bibliographic data and their corresponding PDF files if exit.

* Option: **pmids=<PubMed ID>,<PubMedID>,...,<PubMed ID>**

* Return

**{**

**"ErrorCode":0,**

**"Done":"<Key>"**

**}**

**3.4. reset**

* Description: delete all the registered bibliographic data and stored PDF files.

* Option: none

* Return

**{**

**"ErrorCode":0,**

**"Done":"<Key>"**

**}**

**3.5. count-registered**

* Description: obtain the number of registered bibliographic data.

* Option: none

* Return

**{**

**"ErrorCode":0,**

**"Count":"<Number of the data>"**

**}**

**3.6. enumerate-registered**

* Description: obtain a list of registered PubMed IDs.

* Option: none

* Return

**{**

**"ErrorCode":0,**

**"Registered":["<PubMed ID>","<PubMed ID>", ... ,"<PubMed ID>"]**

**}**

**3.7. list-recommended**

* Description: obtain recommendations.

* Option1: **type=pubmed**

(Currently, only 'pubmed' can be set to 'type')

Option2: **mode=recent**

(If you want recommendations from up to the three most recent months after publications)

Option3: **pmids=<PubMed ID>,<PubMedID>,...,<PubMed ID>**

Option4: **tags=<TagName>,<TagName>,...,<TagName>**

(If there is 'pmids', it is prioritized)

If neither pmids nor tags are given, recommendation based on all of the registered PubMed IDs is obtained.

* Return

**{**

**"ErrorCode":0,**

**"Access":"<URLtoGetRecommendations>"**

**}**

* Return of HTTP GETting to the specified URL

**[**

**{**

**"SC":<Rank from zero, integer>,**

**"VL_IS_PG":"<Volume, Issue, Pages>",**

**"PDATE":"<Publication date>",**

**"SCORE":"<Significance of the paper, float>",**

**"SNIPPETS":"", (currently empty)**

**"PMID":"<PubMed ID>",**

**"JOURNAL":"<Journal name>",**

**"TITLE":"<Title of the paper>",**

**"DOI":"", (currently empty)**

**"AUTHORS":"<Author names>"**

**},...**

**]**

**3.8. enumerate-recommended**

* Description: synonym of list-recommended.

**3.9. set-tags**

* Description: set tags to a given PubMed ID list.

* Option: **tagdata={**

**"<PubMed ID>":["<TagName>","<TagName>",...,"<TagName>"],**

**"<PubMed ID>":["<TagName>","<TagName>",...,"<TagName>"],**

**...,**

**"<PubMed ID>":["<TagName>","<TagName>",...,"<TagName>"]**

**}**

***Note****: tagdata needs to be in the URL-encoded JSON format, and for each PubMed ID an array of tag names is given.*

* Return

**{**

**"ErrorCode":0,**

**"Done":"<Key>",**

**"Revision":"<RevisionNumber>"**

**}**

RevisionNumber is used to synchronize the data (Integer, and is incremented by one when modification occurs).

**3.10. get-tags**

* Description: obtain tags to a given PubMed ID list.

* Option: **pmids=<PubMed ID>,<PubMedID>,...,<PubMed ID>**

* Return

Same as the tagdata option of 'set-tags'

**3.11. enumerate-tags**

* Description: obtain a list of all pairs of PubMed ID and its tag names.

* Option: none

* Return

Same as the tagdata option of 'set-tags'

**3.12. reset-tags**

* Description: remove tag data of a given PubMed ID list.

* Option: **pmids=<PubMed ID>,<PubMedID>,...,<PubMed ID>**

* Return

**{**

**"ErrorCode":0,**

**"Done":"<Key>",**

**}**

**3.13. put-pdf**

* Description: store a PDF file of a given PubMed ID (Only one ID is allowed).

* Option1: **pmids=<PubMed ID>**

Option2: **pdf_file=<raw data of a PDF file>**

* Return

**{**

**"ErrorCode":0,**

**"Done":"<Key>",**

**}**

**3.14. get-pdf**

* Description: obtain a PDF file of a given PubMed ID (Only one ID is allowed).

* Option: **pmids=<PubMed ID>**

* Return

**<raw data of the specified PDF file>**

**3.15. enumerate-pmids-with-pdf**

* Description: obtain a PubMed ID list each of which has its corresponding PDF file.

* Option: none

* Return

**["<PubMed ID>","<PubMed ID>",...,"<PubMed ID>"]**

**3.16. get-tag-revision**

* Description: obtain a revision number.

* Option: none

* Return

**{**

**"ErrorCode":0,**

**"Revision":"<RevisionNumber>"**

**}**

**3.17. get-scanpdf-url**

* Description: obtain the endpoint URL to get a PubMed ID by analyzing a given PDF file.

* Option: none

* Return

**{"ErrorCode":0,"Access":"<URLtoEndPointOfPDFscan>"}**

How to obtain a PubMed ID of the given PDF file

* Arguments of the endpoint

1 **iwt-command=get-pmid**

2 **key=<Key>**

3 **pdf_file=<raw data of a PDF file>**

* Return

**{**

**"ErrorCode":0,**

**"PMIDs":["<PubMed ID>","<PubMed ID>",...,"<PubMed ID>"]**

**}**

***Note****: if the system could not identify a unique PubMed ID, multiple IDs would be returned.*

***Note****: if something goes wrong, returned JSON data follows the above common responses except for the following.*

* If the bibliographic data has been extracted but could not obtain a PubMed ID

**{**

**"ErrorCode":101,**

**"Reason":"No PMIDs were retrieved."**

**"Error":"<Key>",**

**}**

**3.18. get-rating**

* Description: obtain each rating of a given PubMed ID list.

* Option: **pmids=<PubMed ID>,<PubMedID>,...,<PubMed ID>**

* Return

**{**

**"ErrorCode":0,**

**"Rating":["<Rating>","<Rating>",...,"<Rating>"]**

**}**

***Note****: the order of the ratings conforms to that of the given list.*

**3.19. set-rating**

* Description: set each rating of a given PubMed ID list.

* Option1: **pmids=<PubMed ID>,<PubMedID>,...,<PubMed ID>**

Option2: **rating=<Rating>,<Rating>,...,<Rating>**

* Return

**{**

**"ErrorCode":0,**

**"Done":"<Key>",**

**}**

**3.20. reset-rating**

* Description: reset each rating of a given PubMed ID list.

* Option: **pmids=<PubMed ID>,<PubMedID>,...,<PubMed ID>**

* Return

**{**

**"ErrorCode":0,**

**"Done":"<Key>",**

**}**

###
